# Is timing as critical for repair of dextro-transposition of the great arteries with ventricular septal defect without outflow tract obstruction?

**DOI:** 10.1016/j.xjon.2024.10.015

**Published:** 2024-10-26

**Authors:** Muhammad Faateh, Spencer Hogue, Amir Mehdizadeh-Shrifi, Kevin Kulshrestha, Md Monir Hossain, David G. Lehenbauer, David L.S. Morales, Awais Ashfaq

**Affiliations:** a Division of Cardiovascular Surgery, Department of Surgery, The Heart Institute, Cincinnati Children's Hospital and Medical Center, Cincinnati, Ohio; b Department of Pediatrics, University of Cincinnati College of Medicine, Cincinnati, Ohio; c Department of Biostatistics & Epidemiology, Cincinnati Children's Hospital Medical Center, Cincinnati, Ohio

**Keywords:** arterial switch operation, costs, surgical timing, transposition of great arteries, ventricular septal defect

## Abstract

**Objective:**

We sought to explore the role of timing on outcomes of the arterial switch operation + ventricular septal defect closure.

**Methods:**

Neonates undergoing the arterial switch operation + ventricular septal defect closure from the Pediatric Health Information System database (2004-2022) were identified. Patients with outflow tract obstruction were excluded. Baseline features and outcomes were compared by dividing the cohort by age at the arterial switch operation + ventricular septal defect closure: very early (0-7 days), early (8-14 days), late (15-21 days), and very late (>21 days). A cut-point analysis was performed to identify if an age-cutoff predicted the composite outcome (in-hospital mortality/nonhome discharge/postoperative extracorporeal membrane oxygenation/delayed sternum closure/reoperation due to bleeding).

**Results:**

A total of 1005 patients were identified. The median age at repair was 6 days (interquartile range, 4-9). Repair was performed in the majority of study centers within the patient's first week of life. The distribution was very early in 652 patients (64.9%), early in 247 patients (24.6%), late in 72 patients (7.2%), and very late in 34 patients (3.4%). Late and very late groups had a greater proportion of preterm (6.3% vs 13.8% vs 23.2% vs 26.5%) and low-birthweight (5.8% vs 9% vs 21.9% vs 20%) patients (both *P* < .05). In-hospital mortality was 3.1% and similar among groups (*P* > .05). The identified cutoff was 8 days. In-hospital mortality was similar when comparing 0 to 8 days with more than 8 days groups (20 [2.8%] vs 11 [3.9%], *P* = .38). The more than 8 days group was more likely to develop the composite outcome (69 [24%] vs 125 [17.4%], *P* = .02), which remained significant in the multivariable analysis (adjusted odds ratio, 1.49; 95% CI, 1.02-2.15; *P* = .04). Hospitalization costs were significantly higher in the more than 8 days group ($240,742 vs $183,728, *P* < .001).

**Conclusions:**

This analysis of more than 1000 neonates born with dextro-transposition of the great arteries + ventricular septal defect without outflow tract obstruction revealed that most patients undergo the arterial switch operation + ventricular septal defect closure within the first week of life and had acceptable major outcomes regardless of timing. Earlier arterial switch operation + ventricular septal defect closure may confer an advantage with regard to secondary outcomes and hospitalization costs.

**Figure:**
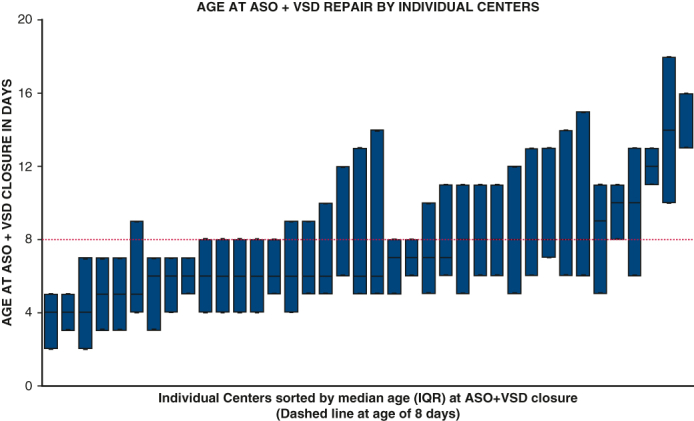
Variation in ASO + VSD timing in individual study centers.

**Central Message**

Most patients undergo ASO + VSD closure within the first week of life. Early versus delayed repair within the first month does not affect mortality; however, repair at more than 1 week may result in worse secondary outcomes.

**Perspective**

The impact of surgical timing of ASO + VSD closure in patients with d-TGA + VSD is not known well because prior reports examining the impact of ASO timing have often combined their outcomes with patients who have d-TGA + IVS. We aim to explore the characteristics, outcomes, and associated costs of early versus delayed ASO + VSD closure.

Dextro-transposition of the great arteries (d-TGA) is associated with ventricular septal defects (VSDs) in 20% to 40% of cases.^1^, ^2^, ^3^, ^4^ Relative to patients with d-TGA with an intact ventricular septum (IVS), the arterial switch operation (ASO) with VSD closure requires longer cardiopulmonary bypass and crossclamp times and is associated with longer postoperative hospital stays, a 2-fold increase in postoperative mortality, and impaired long-term neurological development.^2^, ^3^, ^4^, ^5^, ^6^ There is increasing consensus toward early ASO in patients born with d-TGA + IVS. However, d-TGA + VSD physiology can allow for additional volume loading of the left heart, which increases mixing at the atrial level. An unrestrictive VSD is protective against left ventricular deconditioning. Likely due to these reasons, historically there has been a lower perceived urgency with regard to surgical repair in these patients compared with d-TGA + IVS.^3^^,^^7^ Repair also may be delayed because of patient factors such as delayed presentation, prematurity, or low birth weight. A few prior reports emphasized improved patient outcomes and reduced hospitalization costs for earlier ASO, especially for patients with restrictive VSDs.^8^^,^^9^ However, others have described similar results for early versus delayed ASO + VSD repair.^2^^,^^3^^,^^10^ Most studies examining the impact of ASO + VSD closure timing remain limited to small, single-center patient populations or have not reported outcomes separately for ASO + VSD closure. Moreover, high-risk patients such as low-birthweight and premature patients often have been excluded in these comparisons. Therefore, the present study is a large, multicenter analysis of patient characteristics, institutional variation in timing of early versus delayed repair, and the clinical and financial implications of surgical timing on outcomes of ASO + VSD closure.

## Material and Methods

### Study Population and Data Source

A retrospective analysis of the Pediatric Health Information System (PHIS) database from January 1, 2004, to December 31, 2022, was performed. The PHIS is an administrative database composed of more than 40 major pediatric hospitals across the United States. Detailed information can be accessed at www.childrenshospitals.org. Patients born with a diagnosis of d-TGA + VSD undergoing ASO + VSD closure between the ages of 0 to 30 days were identified. Patients were excluded if they had a coexisting diagnosis of double-outlet right ventricle, outflow tract obstruction, or aortic arch repair. Diagnoses and procedures were identified by International Classification of Diseases 9th and 10th revision codes using the methodology described by Dhillon and colleagues.^11^ The Cincinnati Children's Hospital Institutional Review Board approved this study, and the need for individual patient consent was waived because this was a retrospective analysis of de-identified data (IRB #2018-6837; date of approval: October 29, 2018).

### Covariates and Outcomes

Race and ethnicity were combined into a single categorical variable. Prematurity was defined as gestational age of less than 37 weeks and derived from PHIS-reported gestational age variable, and in case of missing information it was obtained by gestational age International Classification of Diseases codes. Birth weight was similarly derived. Household income is the median household income of a patient's zip code. This was adjusted for inflation (2022 USD) using urban consumer price index. Prostaglandin or inhaled nitric oxide variables indicate the use of these medications at any time preoperatively. The primary outcomes of interest were in-hospital mortality (death or discharge to hospice status) and postoperative extracorporeal membrane oxygenation (ECMO). Secondary outcomes included delayed sternum closure, reoperation for control of bleeding, and nonhome discharge. Nonhome discharge includes discharge to a rehabilitation/short- or long-term acute care or nursing facility, as well as transfers to other hospitals. A binary composite outcome was created that denotes occurrence of any components of the primary or secondary outcomes. A variable containing primary outcomes indicating in-hospital death or postoperative ECMO was also created. Additional outcomes included length of stay, postoperative dialysis use, permanent pacemaker implantation, and adjusted estimated hospitalization costs. Hospitalization costs were derived from charge-to-cost ratios reported by each hospital and adjusted for inflation (2022 USD) using medical consumer price index. The prevalence of concomitant morbidities/diagnoses, including stroke, heart failure, pulmonary hypertension, acute respiratory distress syndrome, necrotizing enterocolitis, hepatic failure, renal failure, and sepsis, was identified. The timing (preoperative vs postoperative) of these variables could not be distinguished because the database only indicates if they were present at the time of admission to hospital or occurred at any point during the hospitalization.

### Statistical Analysis

The entire cohort was divided into 4 subgroups based on age at ASO + VSD closure. The groups were very early (0-7 days), early (8-14 days), late (15-21 days), and very late (>21 days). Baseline characteristics, outcomes, and hospitalization costs were compared by these groups. A cut-point analysis was performed by using a predictive ability metric, plotting the sum of sensitivity and specificity against age (in days) by serially dichotomizing the data by age at surgery (ie, age 0 day vs > 0, age 0-1 day vs > 1, age 0-29 vs 30 days) to identify if a clear cutoff day/range for ASO + VSD closure was a predictor of developing the composite outcome. The cut-point with the highest sum of sensitivity + sensitivity was chosen for the final comparisons.

In addition, subanalyses of outcomes by age at ASO + VSD closure were performed stratified by prematurity (<37 weeks), low birth weight (<2.5 kg), and delayed presentation (admission age >3 days). The median age and interquartile range (IQR) of ASO + VSD closure for individual hospitals were computed to assess variation in timing of ASO by center. Chi-square, *t* test, rank sum, Kruskal–Wallis with pairwise comparisons, and analysis of variance were used to compare baseline characteristics and outcomes as appropriate. Multivariable logistic regression analysis was performed by adjusting for clinically significant predictors of the composite outcome. All analyses were performed using Stata 18.0 (Stata Corp).

## Results

A total of 1005 patients undergoing ASO + VSD were identified. The median age at ASO + VSD closure for the entire cohort was 6 days (IQR, 4-9). Overall, 31 patients (3.1%) experienced in-hospital death. The distribution of patients by age at ASO + VSD closure groups was as follows: very early 652 (64.9%), early 247 (24.6%), late 72 (7.2%), and very late 34 (3.4%). Patients in the late and very late groups were more likely to be preterm (39 [6.3%] vs 33 [13.8%] vs 16 [23.2%] vs 9 [26.5%], respectively, *P* < .001, very early vs all other groups pairwise <.05) and to have low birth weight (35 [5.8%] vs 21 [9%] vs 14 [21.9%] vs 6 [20%], respectively, *P* < .001, all pairwise <.05). The latter groups had a higher proportion of patients with delayed presentation (admission age >3 days) (26 [3.9%] vs 31 [12.6%] vs 24 [33.3%] vs 16 [47.1%], respectively, *P* < .001). Patients across groups were similar in terms of race/ethnicity, gender, insurance, preoperative stroke (on admission), presence of atrial septal defect (ASD), coronary anomalies, or balloon atrial septostomy (all *P* > .05). Detailed baseline comparisons are shown in Table 1.

**Table 1:** Baseline characteristics of patients with dextro-transposition of the great arteries + ventricular septal defect by age at arterial switch operation + ventricular septal defect closure

Variables	Very early (0-7 d) n (%) = 652 (64.9)	Early (8-14 d) n (%) = 247 (24.6)	Late (15-21 d) n (%) = 72 (7.2)	Very late (>21 d) n (%) = 34 (3.4)	*P* value
Median (IQR) age at ASO + VSD closure	5, 3-6 d	10, 8-12 d	18, 16-20 d	25, 23-28 d	-
Mean, SD	4.6, 1.8 d	10.3, 1.9 d	17.7, 2.1 d	25.3, 2.7 d	-
Female gender	197 (30.2)	77 (31.2)	21 (29.2)	17 (50.0)	.11
Race/ethnicity					.57
White	344 (52.8)	127 (51.4)	43 (59.7)	22 (64.7)	
Black	51 (7.8)	24 (9.7)	5 (6.9)	1 (2.9)	
Other	94 (14.4)	43 (17.4)	10 (13.9)	2 (5.9)	
Hispanic	116 (17.8)	43 (17.4)	11 (15.3)	7 (20.6)	
Unknown	47 (7.2)	10 (4.1)	3 (4.2)	2 (5.9)	
Gestational age, wk					<.001
≥37	585 (89.7)	206 (83.4)	53 (73.6)	25 (73.5)	A vs B <.001
33-36	38 (5.8)	29 (11.7)	13 (18.1)	8 (23.5)	A vs C <.001
29-32	1 (0.2)	3 (1.2)	2 (2.8)	1 (2.9)	A vs D <.001
≤28	0 (0)	1 (0.4)	1 (1.4)	0 (0.0)	
Unknown	28 (4.3)	8 (3.2)	3 (4.2)	0 (0.0)	
Birth weight, g					<.001
≥2500	570 (87.4)	212 (85.8)	50 (69.4)	24 (70.6)	A vs B <.001
2000-2499	30 (4.6)	18 (7.3)	11 (15.3)	4 (11.8)	A vs C <.001
<2000	5 (0.8)	3 (1.2)	3 (4.2)	2 (5.9)	A vs D = .003
Unknown	47 (7.2)	14 (5.7)	8 (11.1)	4 (11.8)	
Median age at admission	0, 0-1 d	0, 0-2 d	1, 0-12 d	3, 0-20 d	<.001
Admission age >3 d	26 (3.9)	31 (12.6)	24 (33.3)	16 (47.1)	<.001
Origin of admission					<.001
Birth	171 (26.2)	43 (17.4)	6 (8.3)	3 (8.8)	
Transfer	46 (7.1)	19 (7.7)	14 (19.4)	10 (29.4)	
Outpatient	381 (58.4)	162 (65.6)	40 (55.6)	17 (50.0)	
Unknown	54 (8.3)	23 (9.3)	12 (16.7)	4 (11.8)	
Government/state insurance	273 (43.3)	122 (50.0)	35 (49.3)	15 (44.1)	.31
Median household income (IQR)	$56,859, 43,716-77,216	$52,875, 41,969- 66,534	$53,941, 44,658-68,836	$49,753, 40,086-71,972	.04
Era					.56
2004-2010	300 (46.0)	101 (40.9)	37 (51.4)	12 (35.3)	
2011-2016	206 (31.6)	87 (35.2)	22 (30.6)	12 (35.3)	
2017-2022	146 (22.4)	59 (23.9)	13 (18.1)	10 (29.4)	
PDA	503 (77.2)	210 (85.0)	59 (81.9)	22 (64.7)	.01
ASD	594 (91.1)	222 (89.9)	65 (90.3)	29 (85.3)	.69
Coronary anomalies	70 (10.7)	34 (13.8)	14 (19.4)	3 (8.8)	.13
Preoperative stroke (on admit)	15 (2.3)	12 (4.9)	0 (0)	2 (5.9)	.04
BAS	251 (38.5)	104 (42.1)	30 (41.7)	15 (44.1)	.71
Age at BAS, median, IQR	1, 0-1 d	1, 1-2 d	1, 1-14 d	2, 1-14 d	<.001
ASD repair	551 (84.5)	208 (84.2)	64 (88.9)	30 (88.2)	.78
Preoperative ECMO	2 (0.3)	3 (1.2)	1 (1.4)	0 (0)	.22
Preoperative PGE-1 infusion	525 (85.2)	182 (79.8)	44 (65.7)	19 (57.6)	<.001
Preoperative iNO	43 (7.0)	19 (8.3)	4 (5.9)	3 (9.1)	.82

*IQR*, Interquartile range; *ASO*, arterial switch operation; *VSD*, ventricular septal defect; *PDA*, patent ductus arteriosus; *ASD*, atrial septal defect; *BAS*, balloon atrial septostomy; *ECMO*, extracorporeal membrane oxygenation; *PGE-1*, prostaglandin E-1; *iNO*, inhaled nitric oxide.

### Outcomes Stratified by Repair Age

Patients in all 4 age groups (very early vs early vs late vs very late) had similar in-hospital mortality (17 [2.6%] vs 11 [4.5%] vs 2 [2.8%] vs 1 [2.9%], respectively, *P* = .47) and postoperative ECMO requirement (21 [3.2%] vs 11 [4.5%] vs 4 [5.6%] vs 2 [5.9%], respectively, *P* = .40). Patients in the very late group had the highest proportion of delayed sternum closure (47 [7.2%] vs 23 [9.3%] vs 8 [11.1%] vs 8 [23.5%], respectively, *P* = .007). The composite outcome was lowest in the very early age group (106 [16.3%] vs 61 [24.7%] vs 17 [23.6%] vs 10 [29.4%], respectively, *P* < .001, very early vs early *P* = .004, very early vs late *P* = .115, very early vs very late, respectively, *P* = .046). The postoperative length of stay was similar across groups (*P* = .66). Median hospitalization costs were lowest in the very early group ($138,330 vs $177,376 vs $202,394 vs $202,268, respectively, *P* < .001).

Concomitant diagnoses/outcomes (occurring preoperatively or postoperatively) were as follows: heart failure (108 [16.6%] vs 52 [21.1%] vs 17 [23.6%] vs 12 [35.3%], *P* = .018), stroke (37 [5.7%] vs 26 [10.5%] vs 6 [8.3%] vs 4 [11.8%], *P* = .04), necrotizing enterocolitis (8 [1.2%] vs 3 [1.2%] vs 3 [4.2%] vs 5 [14.7%], *P* < .001), and renal failure (75 [11.5%] vs 37 [14.9%] vs 8 [11.1%] vs 10 [29.4%], respectively, *P* = .015) (Table 2).

**Table 2:** Outcomes by age at arterial switch operation + ventricular septal defect closure

	Very early (0-7 d) n (%) = 652 (64.9)	Early (8-14 d) n (%) = 247 (24.6)	Late (15-21 d) n (%) = 72 (7.2)	Very late (>21 d) n (%) = 34 (3.4)	*P* value
Death	17 (2.6)	11 (4.5)	2 (2.8)	1 (2.9)	.47
Postoperative ECMO	21 (3.2)	11 (4.5)	4 (5.6)	2 (5.9)	.40
Death or ECMO	28 (4.3)	16 (6.5)	5 (6.9)	2 (5.9)	.38
Delayed sternum closure	47 (7.2)	23 (9.3)	8 (11.1)	8 (23.5)	.007
Reoperation for bleeding	28 (4.3)	23 (9.3)	3 (4.2)	2 (5.9)	.03
Composite of the above outcomes	106 (16.3)	61 (24.7)	17 (23.6)	10 (29.4)	<.001 A vs B = .004 A vs C = .115 A vs D = .046
Permanent pacemaker	23 (3.5)	6 (2.4)	2 (2.8)	0 (0)	.77
Postoperative dialysis	11 (1.8)	7 (3.1)	4 (5.9)	0 (0)	.12
Nonhome discharge (survivors)	17 (2.7)	9 (3.8)	3 (4.3)	0 (0)	.59
Total LOS, median, IQR	17, 13-23 d	22, 18-29 d	28, 20-39 d	34, 21-48 d	<.001
Postoperative LOS, median, IQR	13, 9-18 d	12, 9-19 d	15, 9-25 d	12, 9-25 d	.66
Total ICU LOS, median, IQR	13, 10-19 d	18, 15-24 d	25, 12-35 d	21, 10-45 d	<.001
Postoperative ICU LOS, median, IQR	8, 5-14 d	8, 5-13 d	11, 6-19 d	9, 6-21 d	.22
Hospitalization costs, median, IQR	$138,330, 108,257-196,588	$177,376, 137,929-247,703	$202,394, 125,784-346,141	$202,268, 136,146-367,160	<.001 A vs B <.001 A vs C <.001 A vs D = .001
Concomitant outcomes/diagnoses for the entire admission (occurring preoperatively or postoperatively)					
Stroke	37 (5.7)	26 (10.5)	6 (8.3)	4 (11.8)	.04
Heart failure	108 (16.6)	52 (21.1)	17 (23.6)	12 (35.3)	.018
Pulmonary hypertension	9 (1.4)	3 (1.2)	3 (4.2)	0 (0)	.31
ARDS	93 (14.3)	39 (15.8)	13 (18.1)	4 (11.8)	.75
Necrotizing enterocolitis	8 (1.2)	3 (1.2)	3 (4.2)	5 (14.7)	<.001
Hepatic failure	0 (0)	1 (0.4)	0 (0.0)	1 (2.9)	.028
Renal failure	75 (11.5)	37 (14.9)	8 (11.1)	10 (29.4)	.015
Sepsis	14 (2.2)	5 (2.0)	3 (4.2)	0 (0)	.62

*ECMO*, Extracorporeal membrane oxygenation; *LOS*, length of stay; *IQR*, interquartile range; *ICU*, intensive care unit; *ARDS*, acute respiratory distress syndrome.

### Outcomes in Preterm (<37 Weeks), Low-Birthweight (<2.5 kg), and Delayed Presentation (Admission Age >3 Days) Groups

The overall mortality in preterm neonates was 10 of 97 (10.3%) and similar by age at surgery (*P* = .23). The composite outcome in preterm patients was also similar across groups (14 [35.9%] vs 12 [36.4%] vs 5 [31.3%] vs 5 [55.6%], respectively, *P* = .67). Likewise, the mortality and composite outcomes were similar in low-birthweight patients and patients with delayed presentation (all *P* > .05). Patients who were born at term, had normal birth weight, and were admitted within 3 days of birth had similar mortality by age at surgery (*P* = .67); however, there was a lower proportion of patients who had delayed sternum closure or composite outcome in the very early group (both *P* < .05) (Table 3).

**Table 3:** Logistic regression comparing arterial switch operation + ventricular septal defect closure outcomes by age cutoff (0-8 vs >8 days)

	Univariable OR (95% CI)	*P* value	Adjusted OR∗ (95% CI)	*P* value
In-hospital mortality	1.40 (0.66-2.96)	.38	-	-
Postoperative ECMO	1.67 (0.86-3.26)	.13	-	-
Death or ECMO	1.53 (0.85-2.74)	.16	-	-
Delayed sternum closure	1.39 (0.87-2.21)	.17	-	-
Reoperation for bleeding	1.55 (0.89-2.71)	.13	-	-
Nonhome discharge	1.14 (0.52-2.53)	.75	-	-
Composite outcome	1.51 (1.08-2.11)	.015	1.47 (1.02-2.15)	.041

*OR*, Odds ratio; *ECMO*, extracorporeal membrane oxygenation.

∗ Adjusted for age at admission, gestational age, coronary anomaly, patent ductus arteriosus, insurance, and household income.

### Variation in Age at Arterial Switch Operation + Ventricular Septal Defect Closure by Center

**Figure 1:**
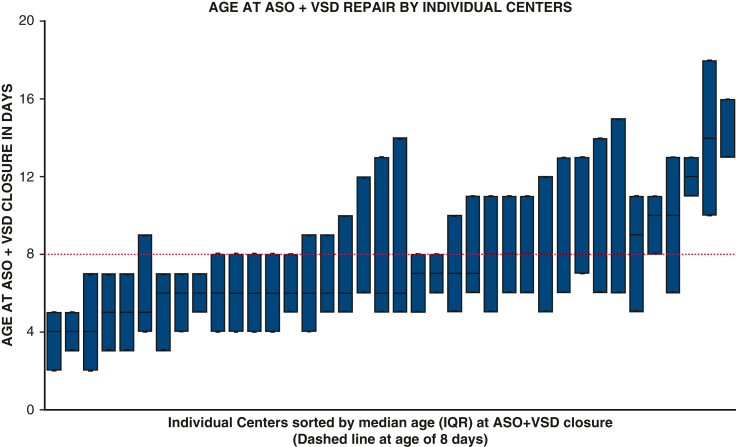
Box-plot showing in median age and IQR of ASO + VSD closure for individual study centers. *IQR*, Interquartile range; *ASO*, arterial switch operation; *VSD*, ventricular septal defect.

ASO + VSD closure was performed in a majority (24/38) of centers in patients with a median age of 7 days or younger. Patients in 8 centers had a median age at surgery of 8 days, and patients in 6 centers had a median age of more than 8 days. Because 6 centers reported less than 5 total cases throughout the study period, they were excluded for this evaluation (Figure 1).

### Comparisons of Outcomes by Age Cutoff Identified by Cut-point Analysis

**Figure 2:**
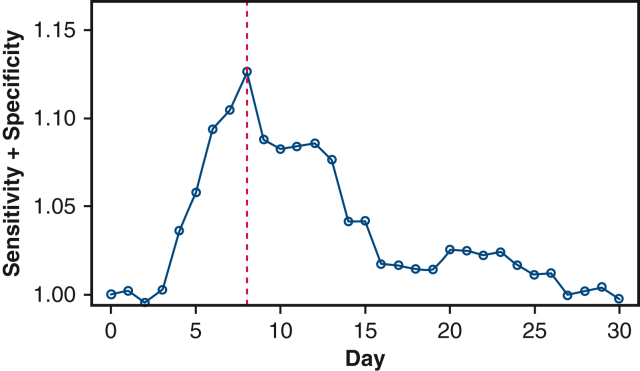
Cut-point analysis revealing 8 days as the cutoff for predicting ASO + VSD outcome (*red line* at 8 days). *ASO*, Arterial switch operation; *VSD*, ventricular septal defect.

The identified cutoff for age at ASO + VSD closure with the maximum sum of sensitivity + specificity was 8 days (Figure 2). Individually, the odds of in-hospital mortality, postoperative ECMO, in-hospital mortality or postoperative ECMO, delayed sternum closure, reoperation for control of bleeding, or nonhome discharge were not statistically significant when ASO + VSD closure was performed after more than 8 days (all *P* > .05). However, the odds of composite outcome comparing ASO + VSD closure age more than 8 days versus 0-8 days was 1.51 (95% CI, 1.08-2.11, *P* = .015). This remained significant in the multivariable regression analysis after adjusting for age at admission, gestational age, coronary anomaly, patent ductus arteriosus, insurance, and household income (OR, 1.47, 95% CI, 1.02-2.15, *P* = .041) (Table 4).

**Table 4:** Subanalysis of arterial switch operation + ventricular septal defect closure outcomes by age in premature, low-birthweight, and delayed presentation patients

	Very early (0-7 d)	Early (8-14 d)	Late (15-21 d)	Very late (>21 d)	*P* value
Premature birth (<37 wk)					
n (%)	39 (40.2)	33 (34.0)	16 (16.5)	9 (9.3)	-
Gestational age, wk					-
33-36	38 (97.4)	29 (87.9)	13 (81.3)	8 (88.9)	
29-32	1 (2.6)	3 (9.1)	2 (12.5)	1 (11.1)	
≤28	0 (0)	1 (3.0)	1 (6.3)	0 (0.0)	
Mortality	4 (10.3)	6 (18.2)	0 (0)	0 (0)	.23
Postoperative ECMO	2 (5.1)	2 (6.1)	0 (0)	0 (0)	1.0
Death or ECMO	5 (12.8)	6 (18.2)	0 (0)	0 (0)	.24
Delayed sternum closure	5 (12.8)	4 (12.1)	2 (12.5)	4 (44.4)	.09
Reoperation for bleeding	3 (7.7)	3 (9.1)	1 (6.3)	1 (11.1)	1.0
Composite outcome	14 (35.9)	12 (36.4)	5 (31.3)	5 (55.6)	.67
Postoperative LOS, median, IQR	17, 13-35 d	19, 12-37 d	18, 9-31 d	22, 15-43 d	.80
Low birth weight (<2.5 kg)					
n (%)	35 (46.1)	21 (27.6)	14 (18.4)	6 (7.9)	-
Birth weight, g					-
2000-2499	30 (85.7)	18 (85.7)	11 (78.6)	4 (66.7)	
<2000	5 (14.3)	3 (14.3)	3 (21.4)	2 (33.3)	
Mortality	4 (11.4)	5 (23.8)	0 (0)	0 (0)	.19
Postoperative ECMO	4 (11.4)	1 (4.8)	0 (0)	0 (0)	.67
Death or ECMO	6 (17.1)	5 (23.8)	0 (0)	0 (0)	.19
Delayed sternum closure	3 (8.6)	4 (19.1)	2 (14.3)	2 (33.3)	.38
Reoperation for bleeding	3 (8.6)	2 (9.5)	0 (0)	0 (0)	.80
Composite outcome	12 (34.3)	11 (52.4)	3 (21.4)	2 (33.3)	.32
Postoperative LOS, median, IQR	17, 12-37 d	16, 11-35 d	16, 12-18 d	34, 22-86 d	.15
Delayed presentation (age at admission >3 d)					
n (%)	26 (26.8)	31 (31.9)	24 (24.7)	16 (16.5)	-
Admission age in d	4, 4-5 d	7, 5-8 d	14, 12-17 d	20, 14-23 d	-
Mortality	1 (3.9)	0 (0)	1 (4.2)	1 (6.3)	.48
Postoperative ECMO	2 (7.7)	0 (0.0)	2 (8.3)	1 (6.3)	.36
Death or ECMO	2 (7.7)	0 (0)	3 (12.5)	1 (6.3)	.19
Delayed sternum closure	2 (7.7)	1 (3.3)	0 (0)	1 (6.3)	.55
Reoperation for bleeding	1 (3.9)	2 (6.5)	2 (8.3)	1 (6.3)	.94
Composite outcome	5 (19.2)	6 (19.4)	7 (29.2)	3 (18.8)	.83
Postoperative LOS, median, IQR	10, 9-15 d	9, 7-12 d	12, 7-18 d	11, 8, 14 d	.39
Term (≥37 wk), normal birth weight (≥2.5 kg) with admission age ≤3 d					
n (%)	509 (71.6)	165 (23.2)	26 (3.7)	11 (1.6)	-
Mortality	9 (1.8)	3 (1.8)	1 (3.9)	0 (0)	.67
Postoperative ECMO	14 (2.8)	7 (4.2)	2 (7.7)	1 (9.1)	.14
Death or ECMO	17 (3.3)	7 (4.2)	2 (7.7)	1 (9.1)	.25
Delayed sternum closure	34 (6.7)	16 (9.7)	4 (15.4)	3 (27.3)	.025
Reoperation for bleeding	21 (4.1)	16 (9.7)	1 (3.9)	1 (9.1)	.041
Composite outcome	74 (14.5)	36 (21.8)	6 (23.1)	3 (27.3)	.07
Postoperative LOS, median, IQR	13, 9-18 d	12, 9-18 d	19, 9-25 d	13, 9-38 d	.17

*ECMO*, Extracorporeal membrane oxygenation; *LOS*, length of stay; *IQR*, interquartile range.

## Discussion

To our knowledge, the present multi-institutional study is the largest report of d-TGA + VSD as an isolated cohort, including more than 1000 ASO + VSD closures. Most patients underwent ASO + VSD closure early within the first week of life. Patients who underwent delayed repair were more likely to be premature, to have low birth weight, or to have delayed presentation. Overall, in-hospital mortality and primary outcomes were similar in all 4 groups. However, earlier repair may have advantages with regard to secondary outcomes and healthcare use burden.

Most centers in the present study performed ASO + VSD closure within the first week. This represents a departure from the traditional approach of delayed repair for d-TGA + VSD and may be a result of contemporary literature that has generally encouraged performance of ASO as early as possible. Anderson and colleagues^4^ reported a 40% increase in risk each day ASO is delayed (>3 days) from a cohort of 140 ASOs, of which 35 (25%) had VSD closure.^4^ Pletzer and colleagues^8^ reported adverse outcomes with increasing surgical age of ASO + VSD closure in infants. Ahlström and colleagues^9^ did not find significant differences in outcomes of delayed ASO (>7 days) from a cohort of 241 ASOs, of which 83 had d-TGA + VSD. It is important to consider that most of these aforementioned studies included d-TGA + IVS, which is more sensitive to timing. Our results suggest that surgical timing (within the first month) does not play a major role in mortality in patients with d-TGA + VSD; however, earlier repair could be beneficial for improving secondary outcomes, such as the need for delayed sternal closure and hospitalization costs.

In patients undergoing ASO, the requirement of postoperative ECMO, delayed sternal closure, and reoperation for bleeding is likely multifactorial.^12^^,^^13^ In the present study, however, mortality and ECMO were similar by surgical timing, despite a greater proportion of preterm and low-birthweight patients in the late groups. The major drivers of delayed repair were patient factors such as prematurity, low birth weight, and delayed presentation. In addition, transfer from an outside hospital and lower household income are likely surrogates for lack of prenatal diagnosis. Prior studies reporting outcomes by the timing of ASO + VSD closure have often excluded patients born prematurely or with low birth weight.^1^^,^^4^ One report suggested that delays in ASO in low-birthweight patients may be unnecessary.^14^ In the present study, the mortality, length of stay, and total hospitalization costs were similar by surgical timing in preterm and low-birthweight patients.

Complications, including stroke, heart failure, necrotizing enterocolitis, and renal failure, were all significantly higher in the late ASO + VSD closure group. Lim and colleagues^2^ highlighted a significant association between the presence of d-TGA + VSD and older age at surgical repair with postoperative neurodevelopmental dysfunction from a cohort of 45 patients with d-TGA, of whom 11 underwent ASO + VSD closure. Moreover, the general presentation of a hemodynamically significant VSD with d-TGA has demonstrated elevated risks for congestive heart failure compounded by increased durations between birth and ASO.^7^^,^^15^ However, we were unable to discern from this study when these were diagnosed during their admission because of limitations of the database.

### Limitations

This study has some limitations. These are related to its retrospective nature and analysis of administrative database. Operative variables such as bypass or crossclamp times, VSD size, or location were not available. We were unable to distinguish or adjust for the timing of certain key factors such as failure of organ systems. Although we used a regression model and performed a stratified analysis, the exact factors and decision-making that may have led to delay were unavailable to be determined from this database. It is challenging to completely uncouple surgical timing from individual patient characteristics that may have led to delays. We were unable to assess the role of surgical timing by corrected gestational age. We do not report outcomes beyond the in-hospital period and may have missed a few early deaths within the perioperative period. The strengths of this study are its large sample size drawn from multiple centers across the United States, availability of financial data that offer a broad picture of contemporary ASO + VSD outcomes, and insight into potential etiologies of early versus late surgical timing of repair.

## Conclusions

This analysis of more than 1000 neonates born with d-TGA with a VSD without outflow tract obstruction revealed similar post–ASO + VSD closure in-hospital mortality within the first 30 days of life. In the contemporary era, surgery was performed at most study centers within the first week of the patient's life. Reasons for delay beyond this period included prematurity, low birth weight (<2.5 kg), and delayed presentation (>3 days of birth). Earlier repair may be advantageous with respect to secondary outcomes and healthcare use burden.

### Webcast

You can watch a Webcast of this AATS meeting presentation by going to: https://www.aats.org/resources/early-versus-late-arterial-swi-7314.

**Figure:**
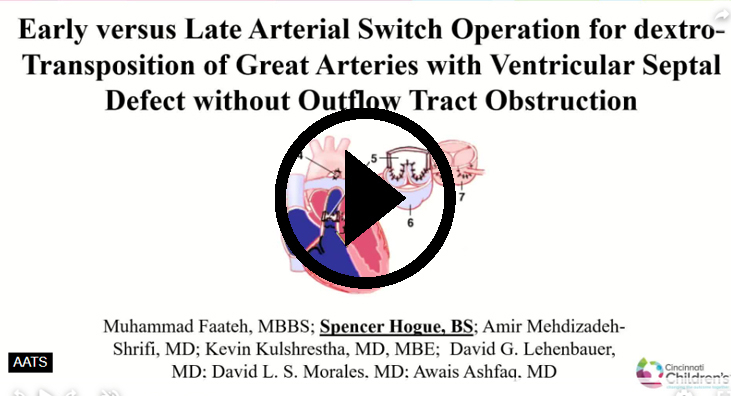

## Conflict of Interest Statement

A.A.: Principal Investigator for Pyrames. D.L.S.M.: Abbott Vascular, Aziyo, Xeltis BV, Berlin Heart, SynCardia Systems (consulting/advisory). All other authors reported no conflicts of interest.

The *Journal* policy requires editors and reviewers to disclose conflicts of interest and to decline handling or reviewing manuscripts for which they may have a conflict of interest. The editors and reviewers of this article have no conflicts of interest.
